# Identification of psoriatic arthritis mediators in synovial fluid by quantitative mass spectrometry

**DOI:** 10.1186/1559-0275-11-27

**Published:** 2014-07-01

**Authors:** Daniela Cretu, Ioannis Prassas, Punit Saraon, Ihor Batruch, Rajiv Gandhi, Eleftherios P Diamandis, Vinod Chandran

**Affiliations:** 1 Department of Laboratory Medicine and Pathobiology, University of Toronto, Toronto, Ontario, Canada; 2 Department of Pathology and Laboratory Medicine, Mount Sinai Hospital, Toronto, Ontario, Canada; 3 Arthritis Program, Toronto Western Hospital, University Health Network, 399 Bathurst Street, Room 1E 416, Toronto, ON M5T 2S8, Canada; 4 Division of Orthopaedic Surgery, Department of Surgery, University of Toronto, Toronto, Canada; 5 Department of Clinical Biochemistry, University Health Network, Toronto, Ontario, Canada; 6 Psoriatic Arthritis Program, Centre for Prognosis Studies in the Rheumatic Diseases, Toronto Western Hospital, Toronto, Ontario, Canada; 7 Division of Rheumatology, Department of Medicine, University of Toronto, Toronto, Ontario, Canada

**Keywords:** Psoriatic arthritis, Early osteoarthritis, Proteomics, Mass spectrometry, Synovial fluid, Selected reaction monitoring assays, Proteins, Mediators

## Abstract

**Background:**

Synovial fluid (SF) is a dynamic reservoir for proteins originating from the synovial membrane, cartilage, and plasma, and may therefore reflect the pathophysiological conditions that give rise to arthritis. Our goal was to identify and quantify protein mediators of psoriatic arthritis (PsA) in SF.

**Methods:**

Age and gender-matched pooled SF samples from 10 PsA and 10 controls [early osteoarthritis (OA)], were subjected to label-free quantitative proteomics using liquid chromatography coupled to mass spectrometry (LC-MS/MS), to identify differentially expressed proteins based on the ratios of the extracted ion current of each protein between the two groups. Pathway analysis and public database searches were conducted to ensure these proteins held relevance to PsA. Multiplexed selected reaction monitoring (SRM) assays were then utilized to confirm the elevated proteins in the discovery samples and in an independent set of samples from patients with PsA and controls.

**Results:**

We determined that 137 proteins were differentially expressed between PsA and control SF, and 44 were upregulated. The pathways associated with these proteins were acute-phase response signalling, granulocyte adhesion and diapedesis, and production of nitric oxide and reactive oxygen species in macrophages. The expression of 12 proteins was subsequently quantified using SRM assays.

**Conclusions:**

Our in-depth proteomic analysis of the PSA SF proteome identified 12 proteins which were significantly elevated in PsA SF compared to early OA SF. These proteins may be linked to the pathogenesis of PsA, as well serve as putative biomarkers and/or therapeutic targets for this disease.

## Background

Psoriatic arthritis (PsA) is an inflammatory arthritis distinguished by bone resorption and periarticular new bone formation, and bears its name from its association with the cutaneous disease, psoriasis [1]. PsA occurs in up to 30% of psoriasis patients and in 85% of cases, psoriasis precedes or occurs simultaneously with PsA [2]. PsA has a predicted prevalence of 0.16 to 0.25% in the general population, and is a complex, potentially disabling musculoskeletal disorder often arising early in age. Patients with PsA are also associated with an increased risk of co-morbidities, such as obesity, metabolic syndrome, diabetes, and cardiovascular disease [2,3]. The etiology of psoriasis and PsA remains unclear, but studies indicate that interaction between multiple genetic components and environmental factors are important in the disease pathogenesis [4-6]. It is proposed that environmental factors such as infections by streptococci [7] or articular trauma [8], may trigger immunological alterations in genetically predisposed individuals [4,9] that play important roles in the appearance of both skin and articular disease. From the immunological point of view, changes are observed in both innate and adaptive immunity. Undoubtedly, the identification of key PsA mediators will not only provide valuable information towards a deeper understanding of the molecular basis of the disease, but it might also uncover important PsA biomarkers potentially useful for clinical follow-up and response to treatment.

Mass spectrometry (MS)-based proteomic approaches are well-suited for the discovery of protein mediators of disease. Early studies relied on qualitative (identity-based) analysis, and performance was depended mainly on the sensitivity of the available MS platforms and sample processing prior to MS-analysis [10,11]. More recently, semi-quantitative and quantitative comparisons of protein relative abundance are the preferred methods for identifying differentially expressed proteins [12]. In many cases, chemical or metabolic labeling of samples prior to analysis has been utilized for quantitation, but it has been associated with technical challenges [13,14]. Label-free quantification (LFQ) methods have also been recently optimized, in which quantification is based on the differential peak intensity [extracted ion current (XIC)] of the peptides in each MS scan [15,16].

LFQ quantitative proteomics presents a robust means for obtaining proteome profiles of virtually any biological material [15,16]. Human plasma represents a diverse proteome and is an excellent source for protein mediators of disease, but proteins secreted by adjacent tissues are diluted in blood, and are often undetectable by current MS methods [14]. To circumvent this issue, attention has been focused on proximal fluids, such as ascites [17], and seminal fluid [18] to search for tissue-associated markers. For instance, in the case of PsA, synovial fluid (SF) represents an interesting source of PsA-related proteins secreted by the synovium, ligament, meniscus, articular cartilage, and joint capsule [19]. Moreover, it is well known, that there is an exchange of proteins between SF and the systemic circulation through the synovial lymphatics and vasculature [19]. In support of this, we have demonstrated that proteins elevated in the SF of PsA patients, are likewise upregulated at the serum level [20]. Consequently, we decided to focus on SF for the discovery of key PsA mediators.

In the present study, we performed label-free MS quantitation of SF proteins from PsA and early osteoarthritis (OA). Using a highly sensitive and specific MS-based approach, we confirmed the elevation of specific elevated proteins in an independent set of samples from patients with PsA. These observations may shed new light on the pathogenesis of PsA, offer insights into disease progression, and might reveal potential PsA biomarkers.

## Results

### Delineating the PsA SF proteome

Our LC-MS/MS analysis of SF identified and quantified 443 proteins, which were present in at least two of the three technical replicates representative of each PsA and EOA pool. Of these, 137 proteins were differentially regulated (2.0 < XIC ratio < 0.8) between the PsA and early OA SF, as shown in Additional file 1: Table S1. A total of 44 proteins were upregulated with a PsA/OA ratio greater than 2, while 93 proteins were downregulated, with a ratio less than 0.8. The IPA software was used to identify dysregulated functional pathways associated with both the upregulated and downregulated proteins in the PsA SF proteome. The top five molecular and cellular functions were, cell-to-cell signalling and interaction, cell movement, antigen presentation, cell cycle, and cell morphology, some of which have been shown to be attributes of PsA [21]. The top canonical pathways associated with these proteins were acute-phase response signalling, granulocyte adhesion and diapedesis, and production of nitric oxide and reactive oxygen species in macrophages (Table 1). While some of the functions were similar, the downregulated proteins were less associated with inflammatory processes (Additional file 1: Table S2). Since our ultimate goal is to identify candidate biomarkers for PsA, we decided to focus on proteins that were overexpressed in PsA SF.

**Table 1:** Summary of Ingenuity Pathway Analysis (IPA)-generated functional pathways and diseases associated with elevated proteins identified from PsA SF

**IPA**	**Number of components identified**	**Proteins**	**P-Value**
**Diseases and disorders**			
Connective tissue disorders	8	ACTA1, DEFA1, IGHM, PLS2, MMP1, MMP3, MPO, S100A9	1.21E-07
Inflammatory disease	8	ACTA1, DEFA1, IGHM, PLS2, MMP1, MMP3, MPO, S100A9	1.21E-07
Skeletal and muscular disorders	8	ACTA1, DEFA1, IGHM, PLS2, MMP1, MMP3, MPO, S100A9	1.21E-07
Inflammatory response	10	CTSG, FGA, PLS2, S100A9, APCS, MPO, SAA1, DEFA1, ORM1, MMP1	2.86E-06
Immunological disease	5	ACTA1, DEFA1, PLS2, MPO, S100A9	1.04E-04
**Molecular and cellular functions**			
Cell-to-cell signaling and interaction	11	CTSG, FGA, PLS2, S100A9, MPO, ORM1, DEFA1, APCS, APOC1, SAA1, MMP1	2.86E-06
Cellular movement	8	CTSG, DEFA1, SAA1, PLS2, MMP1, MMP3, S100A9, PFN1	5.69E-05
Antigen presentation	1	PLS2	2.11E-03
Cell cycle	1	PFN1	2.11E-03
Cell morphology	2	PLS2, PFN1	2.11E-03
**Physiological system development and function**			
Hematological system development and function	10	CTSG, FGA, PLS2, S100A9, MPO, ORM1, DEFA1, SAA1, APCS, IGHM	2.86E-06
Immune cell trafficking	10	CTSG, FGA, PLS2, S100A9, MPO, ORM1, DEFA1, SAA1, MMP1, APCS	2.86E-06
Tissue development	8	CTSG, FGA, PLS2, S100A9, MPO, ORM1, SAA1, ACTA1	2.86E-06
Cell-mediated immune response	3	CTSG, DEFA1, PLS2	1.51E-03
Organismal survival	2	ACTB, PLS2	3.33E-03
**Top canonical pathways**			
LXR/RXR activation	5	APOB, APOC1, FGA, ORM1, SAA1	2.70E-06
Acute phase response signaling	5	APCS, C4BP, FGA, ORM1, SAA1	1.53E-05
Clathirin-mediated endocytosis signaling	5	ACTA1, ACTB, APOB, APOC1, ORM1	2.61E-05
Granulocyte adhesion and diapedesis	4	ACTA1, ACTB, MMP1, MMP3	3.53E-04
Production of nitric oxide and reactive oxygen species in macrophages	4	APOB, APOC1, MPO, ORM1	3.09E-04

According to gene ontology functional annotation, the majority of these proteins were extracellular, plasma membrane-associated, or proteins with unknown localization (Additional file 1: Figure S1), consistent with the fact that SF is a proximal fluid, and most of the proteins are likely shed or secreted by chondrocytes, synoviocytes, and inflammatory cells which come in direct contact with this biological fluid [14]. Since inflammation-driven cell death naturally occurs during PsA [22], this could lead to the release of cytosolic proteins into the SF, therefore the cytosolic proteins we identified also hold biological importance. We focused on proteins displaying strong expression in skin, bone, and immune regulatory cells (Additional file 1: Table S3), but excluded immunoglobulins from further analysis. This reduced our list to 20 proteins, from which, high abundance serum proteins, as identified using the Plasma Proteome Database, were excluded (SAA1, APCS, APOC1), as we assumed that these were most likely serum contaminants from the joint aspiration or arthroscopic procedure. This filtering yielded a final set of 17 proteins (Table 2), which we deemed likely to be associated with PsA. The validity of our discovery approach was further enhanced by the discovery of previously investigated PsA- relevant proteins (CRP, MMP3, S100A9) [23,24].

**Table 2:** Fold change (FC) of seventeen elevated and two housekeeping proteins in PsA SF and the corresponding peptides

**Protein description**	**Gene name**	**PsA:OA FC**	**Corresponding SRM peptides**
Orosomucoid 1	ORM1	2.5	WFYIASAFR
Cathepsin G	CTSG	6.7	NVNPVALPR
Profilin 1	PFN1	9.0	TLVLLMGK
Histone 4	H4	8.6	VFLENVIR
Histone 2A type I A	H2AFX	9.7	NDEELNK
Brain acid soluble protein 1	BASP1	7.4	ESEPQAAAEPAEAK
22 kDa interstitial collagenase	MMP1	6.1	DIYSSFGFPR
Leukocyte elastase inhibitor	SERPINB1	4.3	LGVQDLFNSSK
Myeloperoxidase	MPO	3.9	IGLDLPALNMQR
Plastin 2	PLS2	3.9	NWMNSLGVNPR
Galectin-3-binding protein	M2BP	3.8	AVDTWSWGER
C4b-binding protein	C4BP	2.1	YTCLPGYVR
C-reactive protein	CRP	18.7	ESDTSYVSLK
Protein S100A9	S100A9	18.9	LTWASHEK
Stromelysin 1	MMP3	10.6	VWEEVTPLTFSR
Neutrophil alpha defensin 1	DEFA1	13.1	IPACIAGER
CD5-like protein	CD5L	3.0	IWLDNVR
Leucine-rich alpha-2-glycoprotein 1^*^	LRG1	1.0	DLLLPQPDLR
			GQTLLAVAK
Complement Factor I^*^	CFI	1.0	FSVSLK
			YTHLSCDK

^*^Denotes the housekeeping proteins, and the corresponding peptides.

### SRM verification of putative mediators in individual SF samples

**Figure 1:**
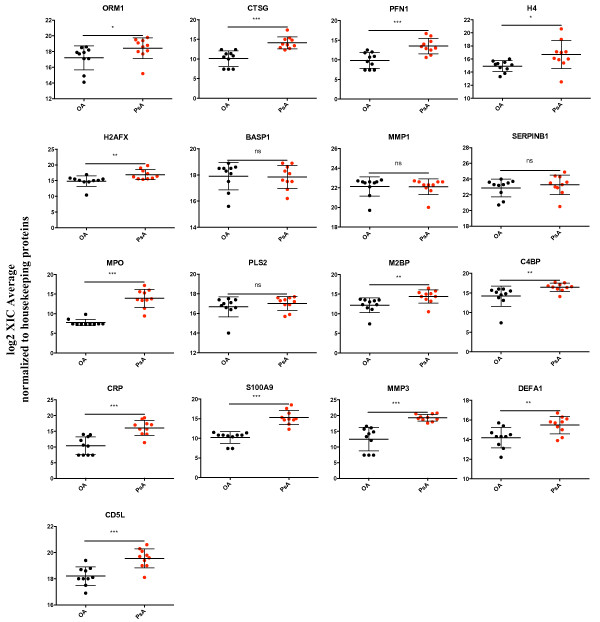
**Verification of elevated proteins in PsA synovial fluid (Set I) by selected reaction monitoring assays, normalized against housekeeping proteins.** Symbols represent SF samples from individual subjects; horizontal lines depict the mean, and vertical lines the standard deviation. ****Indicates P < 0.0001; ***P < 0.001; **P < 0.01; *P < 0.05; ns: non-significant.

To verify the differences in protein expression between PsA and early OA SF samples, as identified by LC-MS/MS, we developed multiplexed selected reaction monitoring assays. Reactions were developed for 17 peptides representative of the 17 proteins with increased expression in the SF of PsA patients, as well as 4 peptides representing the 2 housekeeping proteins that had unchanged expression between PsA and OA SF (Table 2). Consistent with our LC-MS/MS analysis of the pooled samples, overexpression of 13 out of these 17 proteins was also verified in the individual PsA SF samples (Set I), as compared to the early OA SF (Figure 1). Each sample consisted of two technical replicates. CRP, MMP3, and S100A9, were amongst these 13 verified proteins. The mean fold change of each protein and the associated P-values are depicted in Additional file 1: Table S4.

**Figure 2:**
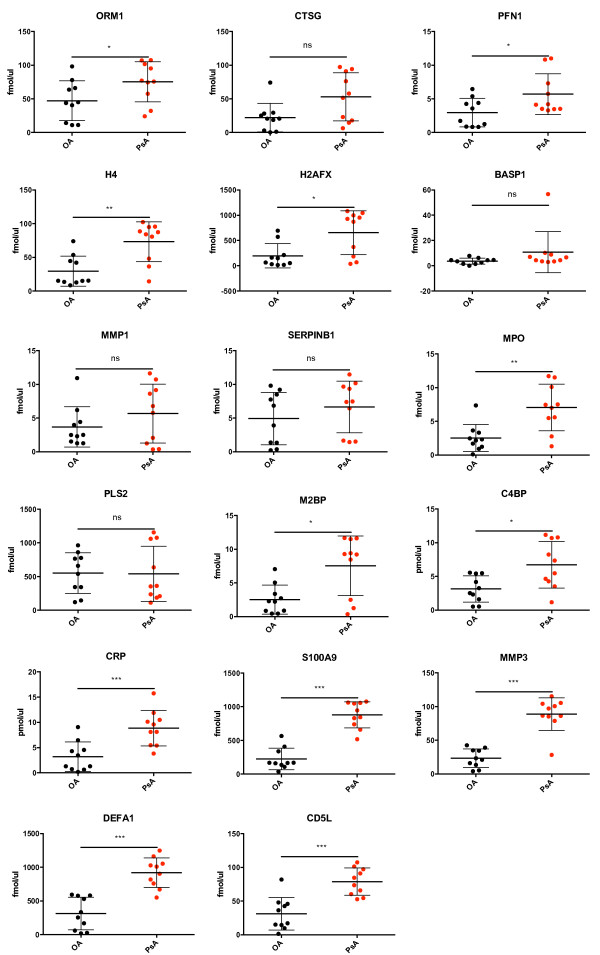
**Verification and concentration of elevated proteins in PsA synovial fluid (Set II) by selected reaction monitoring assays, normalized against heavy-labeled peptides.** Symbols represent SF samples from individual subjects; horizontal lines depict the mean, and vertical lines the standard deviation. ****Indicates P < 0.0001; ***P < 0.001; **P < 0.01; *P < 0.05; ns: non-significant.

Additionally, we also confirmed the elevation of 12 out of the 17 proteins, in an independent set of 10 PsA, and 10 early OA SF samples (Set II). In this case, we utilized heavy-labelled peptides in order to obtain an absolute concentration of these peptides in SF (Figure 2). The proteins included EPO, M2BP, DEFA1, H4, H2AFX, ORM1, CD5L, PFN1, and C4BP, as well as our positive controls MMP3, S100A9, and CRP. The mean fold change and P-values corresponding to each protein from SF set II are also depicted in Additional file 1: Table S4.

## Discussion

In the present study, we performed proteomic analysis of SF, and identified 137 proteins that were differentially expressed between PsA and OA SF. Since one of our future goals is to investigate these proteins as serum biomarkers, we chose to focus only on the 44 upregulated proteins. Moreover, these proteins were reduced to 17 promising candidates that are likely produced by the synovial membrane, cartilage, or surrounding cells, and are secreted into the synovial fluid compartment. The elevation of 12 of these proteins was confirmed in an independent SF cohort, using SRM assays, in order to provide sensitive and specific quantification. As an internal validation of our approach, we re-discovered a number of proteins previously known to be involved in the context of PsA or psoriasis progression. For example, our top 3 up-regulated proteins (based on fold change)- S100A9 [23], MMP3, and CRP [24]- have been extensively studied in the context of PsA.

Psoriasis is a T-cell driven disease that causes epidermal hyperplasia, with an influx of autoimmune effector cells including monocytes, neutrophils, and dendritic cells [25]. Erythema is also an important feature of psoriasis and is caused by increased growth and dilation of superficial blood vessels in the skin [25]. Joint and synovial findings in PsA are T-cell driven, as in the skin, and the prominent hyperplasia of blood vessels in skin is echoed in the synovial membrane [26]. PsA also causes hyper proliferation of the lining cells of the synovium, resulting in the formation of invasive, inflammatory tissue [1,21,27]. These aspects are reflected in the current study, through the identification of upregulated proteins belonging to I) acute-phase response signalling, II) granulocyte adhesion and diapedesis, III) production of nitric oxide and reactive oxygen species in macrophage pathways, and IV) cell-to-cell signalling and interaction, cell movement, and antigen presentation.

One of these proteins, alpha defensin 1 (DEFA1), is secreted by neutrophils in response to various antigens. Elevated levels have been associated with inflammatory activity in both rheumatoid arthritis (RA) [28] and psoriasis [29]. Additionally, antimicrobial defense mechanisms mediated by DEFA1 have also been described in inflammatory synovium [30]. Although a minor inflammatory process is present in OA synovial tissue, PsA patients demonstrate a more aggressive state of synovial tissue inflammation compared with OA patients, therefore elevation in DEFA1 may be a good indicator of PsA pathogenesis.

The enzyme myeloperoxidase (MPO) also seems to play a role in the joint inflammation associated with PsA, as we have determined that it is elevated in PsA SF. MPO is the predominant protein present in primary granules of circulating polymorphonuclear cells. It is a member of the human peroxidase family, heme-containing enzymes that play a role in host defence against infection, and is the major enzymatic source of leukocyte-generated oxidants, released by activated neutrophils and used as a marker of leukocyte recruitment and function and subsequent inflammation [31]. Although not specifically in PsA, in the context of arthritis, MPO has been shown to be elevated in SF and serum derived from RA patients [31], and has been linked to the maintenance of oxidative stress in both psoriasis [32] and RA [33]. Since biological similarities between PsA and RA have been previously described in some disease processes, especially among PsA patients with polyarticular peripheral arthritis [1,27], increased expression of MPO may also represent an important mediator of PsA.

Furthermore, CD5-like protein (CD5L) has been described as an alternative ligand for CD5, a lymphoid-specific membrane glycoprotein that is constitutively expressed in all T cells, but expressed in highest levels in activated T-cells [34,35]. Recently, circulating CD5 levels were shown to be increased in RA and systemic lupus erythematosus [36], and currently we have confirmed the elevation of its ligand, CD5L, in PsA SF. Although the functional relevance of CD5L is unknown, it may play a role in the stimulation and regulation of the immune system [36]. Moreover, a set of recent experiments have provided evidence showing that CD5 stimulation, favors Th17- over Th1-driven polarization of naïve T-cells [37]; the functional relevance of CD5L in this has not been investigated yet. This is relevant since in both psoriatic skin and synovium, while CD8+ T-lymphocytes predominate, the important role played by the Th17 subset of CD4+ T-cells in psoriatic disease has recently also become apparent [38,39]. Together, these findings corroborate our hypothesis, that the proteins we have identified are relevant to PsA and the underlying mechanisms that potentiate this disease.

We also uncovered novel mediators, which have yet to be described in arthritides, which include BASP1, H2AFX, and ORM1. Interestingly, of these, only ORM1 has been previously associated with psoriasis, where ORM1 was increased in the plasma of psoriatic patients [40]. The specific function of this protein has not been determined yet, although it is believed to be involved in aspects of immunosuppression [40]. We speculate that ORM1 may have a protective role in suppressing the immune system to decrease inflammation.

Despite these interesting findings, there are some aspects in the design of this study that need to be further clarified. First, the “control” synovial fluid originated from joints of patients with early OA, and not from healthy individuals. We decided to use these samples for two reasons. One, is that normal SF has proven difficult to obtain, and two, early OA represents an appropriate non-inflammatory comparison, to our inflammatory PsA SF [41].

Second, pooling of samples in the discovery phase of a proteomic study could potentially mask meaningful discrepancies among the different individual SF proteomes. To avoid this, all pool-derived candidates were further examined in all individual samples. While pooling of biological replicates does not allow for statistical comparison, it does produce sufficient sample and increases the likelihood of identifying proteins that are otherwise undetectable (low abundance) in individual samples, therefore allowing a more extensive proteomic coverage of the disease’s heterogeneity. In the current study, we pooled biological replicates and performed SCX fractionation of each pool in triplicate. With the discovery step, we intended to generate a comprehensive SF dataset, in order to identify potential mediators and select SRM candidates. We pooled our biological replicates in order to save instrument time, but also added technical replicates to ensure accuracy of our protein identification. Therefore, by pooling samples as well as quantifying proteins in individual samples as we have done, we have taken advantage of two complementary approaches in order to obtain more meaningful results.

Third, many groups have previously utilized albumin depletion prior to in-gel separation [14], and subsequent proteomic analysis, in order to simplify the SF protein content, and reduce the dynamic range of proteins. We chose to omit this step for the following reasons: I) albumin is a fundamental carrier protein, and therefore, its depletion would result in loss of potentially important proteins from our analysis [14], and II) including a step of immune depletion to our analysis has the potential of increasing the percent error and reducing the reproducibility of our experiments, both of which could increase our false discovery rate [42]. In fact, in a pilot study, where we assessed the protein recovery following albumin depletion, we determined that our protein recovery was 20-40% lower (data not shown).

As discussed previously, quantification of protein levels has been a major challenge in proteomics [13]. We utilized a label-free approach in order to quantify, and identify upregulated proteins in PsA SF. In general, the advantage of label-free approaches over chemical labeling lies in the low cost and the high number of samples that can be easily included in the experiment. Potential disadvantages are lower reproducibility, which may compromise detection of smaller quantitative changes between samples. The lower reproducibility mostly results from the fractionation of peptides prior to LC-MS/MS analysis, and the subsequent pooling of eluted fractions, which can result in unequal/uncontrolled pooling, as was the case in the present study. For example, based on our SCX chromatographic spectra, we noticed several PsA fractions contained higher protein amounts, and pooling of these fractions could hinder the identification of low-abundance proteins; the high-protein fractions were therefore analyzed individually in the PsA group, resulting in the higher number of SCX fractions (15, compared to 10 in early OA,as described in the Methods section). To ensure accuracy and reproducibility of the SCX chromatography, following LC-MS/MS analysis, several peptides were chosen at random, and their occurrence was monitored across fractions corresponding to the PsA and early OA pools. Although the peptide profile was not identical in each fraction, as several peptides in the PsA group spanned multiple fractions, generally the peptide having the highest abundance was found in the same fraction when comparing early OA and PsA groups. Additionally, the reproducibility of LFQ experiments is also largely based on the timeframe of the experiment [14]; therefore, in the present study, we standardized the entire pipeline, from sample collection and processing, to instrument setup and calibration. Despite the shortcomings of the fractionation and pooling strategy, we do verify the overexpression of particular proteins using specific SRM assays, which provides validity to our entire strategy.

## Conclusions

Overall, this study represents the most comprehensive proteomic analysis of PsA synovial fluid, to date. We discovered and verified 12 proteins significantly elevated in PsA SF, compared to early OA SF. Interestingly, the majority of these proteins are part of functional pathways that are known to be dysregulated in psoriasis or PsA. These proteins may serve as potential mediators of the pathogenesis of PsA, and should be further investigated in functional experiments. Also, a large-scale validation of these proteins in serum is essential, in order to investigate these proteins as putative biomarkers and/or therapeutic targets for the detection and treatment of PsA.

## Methods

### SF proteomic analysis

#### Human subjects and clinical samples

The study received institutional review board approval from the University Health Network, and informed consent was obtained from all patients.

For the discovery phase, SF was obtained from 10 cases with PsA (6 males, 4 females; age range 30–76 years), and 10 age- and gender-matched controls (early OA) (Set I). PsA patients had psoriasis and satisfied the CASPAR classification criteria [43]. Inclusion criteria included symptom duration ranging from 1 to 10 years (to capture both relatively early and established disease), and at least one inflamed and accessible large joint. The inflammatory nature of the SF, and the absence of other causes of inflammation, such as infection and/or crystal disease, was confirmed by laboratory investigations.

SF from joints with early OA was obtained during a clinically indicated arthroscopic procedure. Early OA was defined as only a partial thickness cartilage defect in any compartment of the knee, and further defined by a grade I or II lesion by the Outerbridge classification [44].

For the verification (quantification) phase, an independent set of SF samples (Set II) was acquired from 10 PsA patients (7 males, 3 females; age range 21–66 years), and 10 age- and gender-matched early OA controls. Inclusion and exclusion criteria were the same as described above.

#### Pre-analytical sample processing

Synovial fluid samples were stored at −80°C until use. Samples were centrifuged upon thawing at 1800 g for 10 minutes, to remove any cell debris, and the total protein was measured in each sample using the Coomassie (Bradford) total protein assay (Pierce Biotechnology, IL). Equal protein amounts of each SF sample were combined, to obtain two (1 mg total) pools (PsA vs. early OA), which were analyzed in triplicates. Proteins in each pool were denatured using heat (95°C for 10 minutes), reduced with 5 mM dithiothreitol at 60°C for 45 minutes, and alkylated with 15 mM iodoacetamide, in the dark at room temperature for 45 minutes. Sequencing grade trypsin (Promega, WI) was added in a 1:50 (trypsin: protein) ratio, and allowed to digest for 18 hours at 37°C. The samples were subsequently acidified (pH 2) using 1uL of formic acid, to inhibit trypsin activity.

The resulting peptides were then subjected to high-performance liquid chromatography (HPLC) using strong cation exchange (SCX) columns to reduce peptide complexity.

#### HPLC-SCX

Digested samples were diluted 1:2 in mobile phase A SCX buffer (0.26 M formic acid (FA), 10% acetonitrile (ACN); pH 2–3) and loaded directly onto a 500 μL loop connected to a PolySULFOETHYL A column (2.1 mm × 200 mm; 5 μ; 200°A; The Nest Group Inc., MA), containing a silica-based hydrophilic, anionic polymer (poly-2-sulfotheyl aspartamide). An Aglient 1100 HPLC (Agilent Technologies, Germany) system was used for fractionation. A 60-minute gradient was employed with a linear gradient starting at 30 minutes and consisting of mobile phase A and mobile phase B (0.26 M FA, 10% ACN, 1 M ammonium formate; pH-4-5) for elution of peptides (flow rate 200 uL/min). The fractionation was monitored at a wavelength of 280 nm, and performed in triplicate. Fractions were collected every two minutes from 20 to 55 minutes, and those with a low peak absorbance were pooled, resulting in a total of 10–15 fractions per sample (10 fractions for early OA replicates, and 15 fractions for PsA replicates). This amounted to a total of 75 SCX fractions, which were then subjected to liquid chromatographic and tandem mass spectrometric analysis (LC-MS/MS). SCX column and system performance was ensured by running a quality control peptide mixture consisting of 1 ug/uL Alpha Bag Cell peptide, 1 ug/uL Fibrinogen fragment, 5 ug/uL Human ACTH, and 5 ug/uL ACE Inhibitor (American Protein Company, CA) after every sample.

#### LC-MS/MS

The SCX fractions were purified through C-18 OMIX Pipette Tips (Agilent Technologies, Germany), to remove impurities and salts, and eluted in 5 μL of 65% MS buffer B (90% ACN, 0.1% FA, 10% water, 0.02% Trifluoroacetc Acid (TFA)) and 35% MS buffer A (5% ACN, 0.1% FA, 95% water, 0.02% TFA). The samples were diluted to 85 μL in MS buffer A, and injected into a nano-LC system (Proxeon Biosystems, FL) connected online to an LTQ-Orbitrap (Thermo Fisher Scientific, USA). A 90 minute linear gradient reversed phase chromatography using MS buffer A and MS buffer B, was performed at a flow rate of 400 nL/min to resolve petides on a C-18 column (75 uM × 5 cm; Proxeon Biosystems, FL). The MS parameters were: 300 Da minimum mass, 4000 Da maximum mass, automatic precursor charge selection, 10 minimum peaks per MS/MS scan; and 1 minimum scan per group. XCalibur software v.2.0.7 (Thermo Fisher Scientific, USA) was used for data acquisition.

#### Protein identification and quantification

Raw files corresponding to early OA, and PsA data sets were uploaded into MaxQuant v. 1.2.2.2 (http://www.maxquant.org) [45] and searched with Andromeda (built into MaxQuant) [46] against the nonredundant IPI.Human v.3.71 (86, 309 sequences; released March 2010) database, which contains both forward and reverse protein sequences. Search parameters included a fixed carbamidomethylation of cysteines, and variable modifications of methionine oxidation and N-terminal acetylation. Data was initially searched against a “human first search” database with a parent tolerance of 20 ppm and a fragment tolerance of 0.5 Da in order to calculate and adjust the correct parent tolerance to 5 ppm for the search against the IPI.Human fasta file. During the search, the IPI.Human fasta database was randomized and false detection rate (FDR) was set to 1% at the peptide and protein levels. Data was analyzed using “Label-free quantification” checked, and the “Match between runs” interval was set to 2 min. Proteins were identified with a minimum of one unique peptide. “LFQ Intensity” columns corresponding to the extracted ion current (XIC) value of each protein in replicate early OA, and PsA groups were averaged, and used to calculate PsA/early OA ratios (fold change; FC).

#### Bioinformatic analysis

To minimize false positives, we excluded from further analysis any protein with an individual FDR > 0.05. Proteins displaying an XIC value lower than 100,000 were regarded as absent (noise). We also excluded proteins present in only one of the three technical replicates. Averages of the technical replicate XIC values were calculated for each PsA and early OA group, and ratios of PsA/early OA were used to identify deregulated proteins. Upregulated proteins were denoted with a PsA/early OA ratio (FC) greater than 2, while downregulated proteins had a PsA/early OA ratio (FC) less than 0.8. Housekeeping proteins were represented with a PsA/early OA ratio (FC) of approximately 1.0. To determine the possible origin of differentially expressed proteins at the tissue level, and identify the most probable mediators of PsA, gene names were checked against gene (BioGPS (http://biogps.org/#goto=welcome) [47]), and protein (Human Protein Atlas (http://www.proteinatlas.org/) [48]) databases, to identify proteins with strong expression in PsA-associated tissues and cell types (skin, bone, immune cells) [49]. The Plasma Proteome Database (http://www.plasmaproteomedatabase.org/) [50] was employed to identify proteints present in high-abundance in the serum, which could represent potential contaminants. Selected reaction monitoring (SRM) assays were developed for the top upregulated proteins in the PsA group and housekeeping proteins, and relative protein quantification was performed in individual SF samples to confirm their elevation in PsA SF.

#### Network analysis

The list of upregulated proteins identified by LC-MS/MS was analyzed by pathway analysis using the network-building tool, Ingenuity Pathways Analysis (IPA; Ingenuity Systems, http://www.ingenuity.com), as previously described [51].

### Verification of identified proteins using SRM

SRM methods were developed for verification of protein ratios in SF, following our in-house protocols [52].

#### SRM assay development

PeptideAtlas (http://www.peptideatlas.org/) was utilized to select the most commonly observed 3–4 tryptic peptides for the proteins of interest. Their presence was confirmed using our LC-MS/MS identification data. The uniqueness of peptides was verified using the Basic Local Alignment Search Tool (BLAST; https://blast.ncbi.nlm.nih.gov/Blast.cgi). To identify peptide fragments (transitions) to monitor, *in silico* peptide fragmentation was performed using Pinpoint software (Thermo Fisher Scientific, USA) and 5–6 transitions were selected for each peptide. For method optimization, digested pooled samples of SF used in our LC-MS/MS analysis, were loaded onto a C-18 column (Proxeon Biosystems, FL) coupled to a triple quadrupole mass spectrometer (TSQ Vantage; Thermo Fisher Scientific, USA), and approximately 350 transitions were monitored in 6 subsequent runs. Three transitions of the most intense peptides were used for subsequent quantification assays (Table 2).

#### SF sample preparation

Fifty μg of total protein of each SF sample, were denatured by heat, reduced, alkylated, and trypsin-digested as described previously. In the independent SF sample set (Set II), heavy-labelled versions of the peptides of interest (JPT Peptide Technologies, Germany) ranging from 1 to 1000 fmol/μl of sample were also added, as internal standards. Heavy peptides had identical sequences to the endogenous peptides, except the C-terminal lysine or arginine was labeled with ^13^C and ^15^ N. The resulting peptides were purified through C-18 OMIX Pipette Tips (Agilent Technologies, Germany), and eluted in 3 μL of 65% MS buffer B (90% ACN, 0.1% FA, 10% water, 0.02% TFA) and 35% MS buffer A (5% ACN, 0.1% FA, 95% water, 0.02% TFA). Samples were diluted to 40 μL of MS buffer A, randomized, and loaded onto a C-18 column coupled to a triple quadrupole mass spectrometer.

#### SRM assays

SRM assays were developed on a triple-quadrupole mass spectrometer (TSQ Vantage; Thermo Fisher Scientific, USA) using a nanoelectrospray ionization source (nano-ESI, Proxeon Biosystems, FL), as previously described [52]. Briefly, a 60-minute, three-step gradient was used to load peptides onto the column via an EASY-nLC pump (Proxeon Biosystems, FL), and peptides were analyzed by a multiplexed SRM method using the following parameters: predicted CE values, 0.002 m/z scan width, 0.01 s scan time, 0.3 Q1, 0.7 Q3, 1.5 mTorr Q2 pressure and tuned tube lens values. Quantification, in SF set I, was executed after normalization against a set of 4 peptides corresponding to 2 housekeeping proteins (Table 2), to offset technical variations. Each sample was analyzed in duplicate, using a 60-minute method, whereby 63 transitions were monitored. Quantification in SF set II was executed following normalization against the added heavy-labelled peptides, as described earlier. Each sample was analyzed in duplicate, using a 60-minute method, whereby 114 transitions were monitored. Reproducibility of the SRM signal was confirmed by running a quality control solution of 0.1 fmol/μL BSA, every 10 runs.

#### SRM protein quantification

Raw files recorded for each sample were analyzed using Pinpoint software (Thermo Fisher Scientific, USA) [53], and peptide XICs were extracted. Pinpoint was used for identification and visualization of transitions, as well as manual verification of co-elution of heavy and endogenous peptides. In the first SF set, in order to control for technical variation between the samples, XIC corresponding to each endogenous peptide replicate were divided by the XIC corresponding to the average of the two housekeeping proteins. This value was then averaged amongst the two replicate runs, to obtain “XIC Average normalized to housekeeping proteins”. In SF set II, in order to control for technical variation between the samples and obtain a more robust quantitative value for our proteins of interest, the XIC value corresponding to each endogenous peptide, was divided by the XIC value corresponding to each spiked-in heavy peptide, in order to obtain a L:H (light:heavy) ratio. Since we added a known amount of each heavy peptide to our samples prior to analysis, we used the L:H ratio to calculate the relative concentration of each endogenous peptide corresponding to our proteins of interest.

#### Statistical analysis

**Figure 3:**
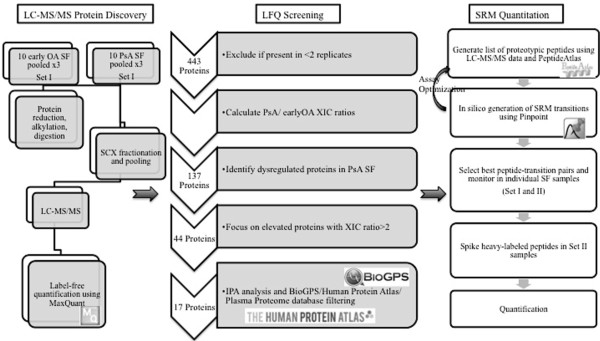
Summary of the experimental design.

Results were analyzed using nonparametric statistics with the Mann–Whitney *U* test. P-values (P) less than 0.05 were considered statistically significant.The overall experimental design is provided in Figure 3.

## Abbreviations

APCS: Amyloid P component; APOC1: Apolipoprotein C1; C4BP: C4b-binding protein; CASPAR: Classification and diagnostic criteria for psoriatic arthritis; CD5L: CD5-like protein; CRP: C-reactive protein; DEFA1: Neutrophil alpha defensin 1; MPO: Myeloperoxidase; H2AFX: Histone 2A type I A; H4: Histone 4; HPLC: High-performance liquid chromatography; LC-MS/MS: liquid chromatographic and tandem mass spectrometric analysis; LFQ: Label free quantification; M2BP: Galectin-3-binding protein; MMP3: Matrix metalloproteinase 3, Stromelysin 1; MS: Mass spectrometry; OA: Osteoarthritis; ORM1: Orosomucoid 1; PFN1: Profilin 1; PsA: Psoriatic arthritis; RA: Rheumatoid arthritis; S100A9: Protein S100A9; SAA1: Serum amyloid A1; SCX: Strong cation exchange; SF: Synovial fluid; SRM: Selected reaction monitoring; XIC: Extracted ion current.

## Competing interests

The authors declare that they have no competing interests.

## Authors’ contributions

DC, VC, and EPD participated in the conception and design of the study. VC provided PsA SF samples. DC and IB performed proteomic analysis, and developed SRM assays. DC, and IB analyzed data and interpreted results. RG provided OA samples. DC, IP, and PS drafted the manuscript. DC, EPD, and VC prepared the final version of the manuscript. All authors read and approved the final manuscript.

## Supplementary Material

Additional file 1: Table S1 Differentially expressed proteins between early OA and PsA groups identified by LC-MS/MS**. Table S2.** Summary of Ingenuity Pathway Analysis (IPA)-generated functional pathways and diseases related to downregulated proteins identified from PsA SF. **Table S3.** Tissue expression of the top 20 elevated proteins identified from PsA SF, through LC-MS/MS. **Table S4.** Fold change (FC) of candidate mediators in Set I and II*. **Figure S1.** Cellular localization of the 44 upregulated proteins based on GO annotation. The numbers depicted in the chart represent the number of proteins with the specified cellular localization. Click here for file
