# Supplementary material for: Identification of psoriatic arthritis mediators in synovial fluid by quantitative mass spectrometry
Source: Clin Proteomics. 2014 Jul 1;11(1):27. doi: 10.1186/1559-0275-11-27 (PMC4108225; doi:10.1186/1559-0275-11-27)
Supplement: Additional file 1: Table S1 — Differentially expressed proteins between early OA and PsA groups identified by LC-MS/MS. Table S2. Summary of Ingenuity Pathway Analysis (IPA)-generated functional pathways and diseases related to downregulated proteins identified from PsA SF. Table S3. Tissue expression of the top 20 elevated proteins identified from PsA SF, through LC-MS/MS. Table S4. Fold change (FC) of candidate mediators in Set I and II*. Figure S1. Cellular localization of the 44 upregulated proteins based on GO annotation. The numbers depicted in the chart represent the number of proteins with the specified cellular localization. [file 1559-0275-11-27-S1.docx]

Table S1. Differentially expressed proteins between early OA and PsA groups identified by LC-MS/MS

| *Upregulated Proteins* | | *Downregulated Proteins* | |
| --- | --- | --- | --- |
| Protein Name | PsA:OA FC | Protein Name | PsA:OA FC |
| A2M | 2.6 | ACAN | 0.2 |
| ACBP | 2.3 | AFM | 0.4 |
| ACTA1 | 3.9 | AMBP | 0.6 |
| ACTB | 3.4 | Anti-RhD monoclonal T125 gamma1 heavy chain | 0.7 |
| ACTBL2 | 3.0 | APOA1 | 0.4 |
| APC2 | 3.2 | APOA4 | 0.6 |
| APCS | 2.4 | AZGP1 | 0.6 |
| APOB | 2.2 | BDK | 0.7 |
| APOBR | 2.2 | BDK | 0.6 |
| APOC1 | 6.3 | BPGF-1 | 0.2 |
| BASP1 | 7.4 | BTD | 0.6 |
| C1QB | 3.5 | C2 | 0.6 |
| C1R | 2.9 | CLU | 0.6 |
| C4B | 4.4 | Cold agglutinin FS-2 L-chain | 0.7 |
| C4BP | 2.2 | COMP | 0.2 |
| CD5L | 3.1 | CRTAC1 | 0.3 |
| CDABP0047 | 6.7 | CRTL1 | 0.2 |
| CLEC3B | 2.2 | ECM1 | 0.6 |
| CP | 2.9 | EFEMP1 | 0.3 |
| CRP | 18.7 | F13A | 0.4 |
| CTSG | 6.7 | F2 | 0.7 |
| DEFA1 | 13.1 | F5-20 | 0.6 |
| FGA | 2.3 | FGFBP2 | 0.3 |
| FGB | 2.8 | FN1 | 0.8 |
| FGG | 3.3 | FN4 | 0.8 |
| H2AFX | 9.7 | GC | 0.6 |
| H4 | 8.6 | GSN | 0.5 |
| IGCJ | 2.5 | HBA1 | 0.4 |
| IGHC | 2.1 | HPX | 0.8 |
| IGHM | 2.1 | IGFALS | 0.7 |
| ITIH3 | 2.1 | IGH | 0.4 |
| ITIH4 | 2.1 | IGHD | 0.7 |
| M2BP | 3.8 | IGHD | 0.8 |
| MMP1 | 6.1 | IGHD | 0.1 |
| MMP3 | 10.6 | IGHFv | 0.4 |
| MPO | 3.9 | FETUB | 0.5 |
| ORM1 | 2.5 | IGHG1 | 0.6 |
| PFN1 | 9.1 | IGHG4 | 0.6 |
| PLS2 | 3.9 | IGHK | 0.8 |
| PZP | 2.6 | F12 | 0.6 |
| S100A9 | 19.4 | IGHK | 0.7 |
| SAA1 | 12.7 | IGHM | 0.7 |
| SERPINB1 | 4.3 | FBNL | 0.1 |
| VL4 | 2.1 | IGHV | 0.6 |
|  |  | IGHV | 0.4 |
|  |  | IGK | 0.6 |
|  |  | IGK | 0.6 |
|  |  | IGKC | 0.5 |
|  |  | IGKC | 0.5 |
|  |  | IGL | 0.3 |
|  |  | ITIH2 | 0.7 |
|  |  | KRT1 | 0.1 |
|  |  | KRT14 | 0.2 |
|  |  | KRT9 | 0.2 |
|  |  | LDC | 0.3 |
|  |  | MHIG | 0.1 |
|  |  | MIH | 0.7 |
|  |  | MMP2 | 0.5 |
|  |  | MSF | 0.4 |
|  |  | PCI | 0.4 |
|  |  | PCOLCE | 0.3 |
|  |  | PEDF | 0.4 |
|  |  | PLG | 0.8 |
|  |  | PLG | 0.4 |
|  |  | Putative Uncharacterized Protein 26 kDa protein | 0.6 |
|  |  | Putative Uncharacterized Protein 26 kDa protein | 0.6 |
|  |  | Putative Uncharacterized Protein 56 kDa protein | 0.6 |
|  |  | Putative Uncharacterized Protein 57 kDa protein | 0.6 |
|  |  | Putative Uncharacterized Protein 59 kDa protein | 0.6 |
|  |  | Putative Uncharacterized Protein cDNA FLJ78387 | 0.7 |
|  |  | Putative Uncharacterized Protein DKFZp686C11235 | 0.7 |
|  |  | Putative Uncharacterized Protein DKFZp686F0970 | 0.6 |
|  |  | Putative Uncharacterized Protein DKFZp686G11190 | 0.7 |
|  |  | Putative Uncharacterized Protein DKFZp686H17246 | 0.4 |
|  |  | Putative Uncharacterized Protein DKFZp686I04196 | 0.6 |
|  |  | Putative Uncharacterized Protein DKFZp686K03196 | 0.7 |
|  |  | Putative Uncharacterized Protein DKFZp686K18196 | 0.7 |
|  |  | Putative Uncharacterized Protein DKFZp686O01196 | 0.7 |
|  |  | Putative Uncharacterized Protein DKFZp686P15220 | 0.6 |
|  |  | SELP | 0.4 |
|  |  | SEPP1 | 0.4 |
|  |  | SERPINA1 | 0.7 |
|  |  | SERPINA4 | 0.5 |
|  |  | SERPINA5 | 0.4 |
|  |  | SERPINA8 | 0.7 |
|  |  | SERPINF1 | 0.4 |
|  |  | SERPINF2 | 0.4 |
|  |  | SNC73 | 0.8 |
|  |  | TSP4 | 0.4 |
|  |  | VHC | 0.6 |
|  |  | VH | 0.7 |
|  |  | VH3 | 0.6 |
|  |  | VH4 | 0.4 |

Table S2. Summary of Ingenuity Pathway Analysis (IPA)-generated functional pathways and diseases related to downregulated proteins identified from PsA SF

| *IPA* | *Number of Components Identified* | *P-Value* |
| --- | --- | --- |
| **Diseases and Disorders** | |  |
| Neurological Disease | 23 | 1.08E-09 |
| Psychological Disorders | 14 | 1.08E-09 |
| Inflammatory Response | 25 | 4.16E-09 |
| Cardiovascular Disease | 19 | 2.35E-08 |
| Organismal Injury and Abnormalities | 28 | 3.26E-08 |
|  |  |  |
| **Molecular and Cellular Functions** | | |
| Cellular Development | 25 | 2.83E-11 |
| Cell-To-Cell Signaling and Interaction | 27 | 7.03E-11 |
| Cellular Growth and Proliferation | 24 | 7.23E-11 |
| Protein Degradation | 13 | 2.57E-09 |
| Protein Synthesis | 16 | 2.57E-09 |
|  |  |  |
| **Physiological System Development and**  **Function** | | |
| Cardiovascular System Development and Function | 19 | 2.83E-11 |
| Organismal Development | 26 | 2.83E-11 |
| Tissue Development | 28 | 2.83E-11 |
| Hematological System Development and Function | 24 | 2.58E-09 |
| Skeletal and Muscular System Development and Function | 18 | 2.62E-08 |
|  |  |  |
| **Top Canonical Pathways** | |  |
| LXR/RXR Activation | 11 | 2.33E-14 |
| Coagulation System | 8 | 3.09E-14 |
| Acute Phase Response Signaling | 11 | 6.94E-13 |
| Hematopoiesis from Pluripotent Stem Cells | 7 | 6.44E-11 |
| Primary Immunodeficiency Signaling | 7 | 7.42E-11 |

Table S3. Tissue expression of the top 20 elevated proteins identified from PsA SF, through LC-MS/MS

|  | *Human Protein Atlas* | | | *BioGPS** | |
| --- | --- | --- | --- | --- | --- |
| Protein Name | Tissue Expression | | | Tissue Expression | |
| C4BP |  | Immune | Bone | Skin | Immune |
| APCS | Skin | Immune |  |  | Immune |
| ORM1 |  | Immune | Bone |  | Immune |
| CD5L |  | Immune |  | Skin | Immune |
| M2BP | Skin |  | Bone | Skin | Immune |
| PLS2 |  | Immune |  |  | Immune |
| MPO |  | Immune |  |  | Immune |
| SERPINB1 |  | Immune |  |  | Immune |
| MMP1 |  |  | Bone |  |  |
| APOC1 | Skin |  |  |  | Immune |
| CTSG |  | Immune |  |  | Immune |
| BASP1 | Skin | Immune |  |  | Immune |
| H4 | Skin | Immune | Bone |  | Immune |
| PFN1 | Skin | Immune |  | Skin | Immune |
| H2AFX | Skin | Immune | Bone | Skin | Immune |
| MMP3 | Skin | Immune | Bone | Skin | Immune |
| SAA1 |  |  |  | Skin |  |
| DEFA1 |  | Immune | Bone |  | Immune |
| CRP | Skin | Immune |  | Skin | Immune |
| S100A9 | Skin |  |  |  | Immune |

*BioGPS does not contain expression data for bone tissue

Table S4. Fold change (FC) of candidate mediators in Set I and II*

|  | ***Set I*** | | ***Set II*** | |
| --- | --- | --- | --- | --- |
| Gene Name | PsA:OA FC | P-Value | PsA:OA FC | P-Value |
| ORM1 | 2.1 | 0.0217 | 1.6 | 0.0487 |
| CTSG | 2.6 | 0.0001 | 2.4 | 0.0887 |
| PFN1 | 2.6 | 0.0006 | 1.9 | 0.0299 |
| H4 | 2.2 | 0.0271 | 2.5 | 0.0015 |
| H2AFX | 2.2 | 0.0022 | 3.3 | 0.0092 |
| BASP1 | 1.9 | 0.8115 | 2.9 | 0.1220 |
| MMP1 | 1.9 | 0.5580 | 1.5 | 0.6182 |
| SERPINB1 | 2.0 | 0.4623 | 1.4 | 0.3298 |
| MPO | 3.5 | 0.0001 | 2.8 | 0.0039 |
| PLS2 | 2.0 | 0.3051 | 1.0 | 0.9365 |
| M2BP | 2.3 | 0.0041 | 3.0 | 0.0048 |
| C4BP | 2.3 | 0.0016 | 2.1 | 0.0105 |
| CRP | 2.9 | 0.0001 | 2.8 | 0.0010 |
| S100A9 | 2.8 | 0.0001 | 3.9 | 0.0010 |
| MMP3 | 2.9 | 0.0001 | 3.8 | 0.0001 |
| DEFA1 | 2.1 | 0.0086 | 2.9 | 0.0001 |
| CD5L | 2.1 | 0.0005 | 2.5 | 0.0002 |

*The description of Sets I and II are given in the experimental methods section.

**Figure S1.** Cellular localization of the 44 upregulated proteins based on GO annotation. The numbers depicted in the chart represent the number of proteins with the specified cellular localization.
